# Flue-cured tobacco confirmed as a reservoir host plant for *Tomato yellow leaf curl virus* by agro-inoculation and *Bemisia tabaci* MED-mediated transmission

**DOI:** 10.1371/journal.pone.0190013

**Published:** 2017-12-22

**Authors:** Meng Li, Xiao-Juan Li, Yun-Lin Su

**Affiliations:** 1 School of Food and Bioengineering, Zhengzhou University of Light Industry, Zhengzhou, Henan, China; 2 Key Laboratory of South China Agricultural Plant Molecular Analysis and Genetic Improvement, South China Botanical Garden, Chinese Academy of Science, Guangzhou, China; Oklahoma State University, UNITED STATES

## Abstract

*Tomato yellow leaf curl virus* (TYLCV) causes great losses in tomato production. In addition to tomato, TYLCV infects many crops or weeds as alternative hosts. These alternative hosts may serve as reservoirs for TYLCV survival and spread. Here, we tested the capability of cultivated, flue-cured tobacco to act as a reservoir host plant for TYLCV. TYLCV DNA was detected in nine flue-cured tobacco cultivars inoculated with an infectious TYLCV clone, although no visible symptoms developed on TYLCV-infected tobacco plants. The percentage of whiteflies with viral DNA increased with an increasing acquisition access period (AAP) and reached 100% after a 12 h AAP on infected tobacco plants. Using infected tobacco plants as virus resources, TYLCV was capable of being transmitted to tobacco and tomato plants by whiteflies, and typical symptoms of TYLCV infection were observed on infected tomato plants but not on infected tobacco plants. Our results suggest that flue-cured tobacco can serve as a reservoir host plant for TYLCV and may play an important role in the spread of TYLCV epidemics in China.

## Introduction

*Tomato yellow leaf curl virus* (TYLCV) causes huge losses in tomato production worldwide [1, 2]. TYLCV is transmitted in a persistent-circulative manner by the whitefly *Bemisia tabaci* (Homoptera: Aleyrodidae) [3], which is a species complex consisting of many closely related cryptic species [4, 5]. The outbreaks of TYLCV epidemics in many countries including China have been found to have a correlation with the global invasion in the past three decades of two devastating cryptic species of the *B*. *tabaci* complex, MEAM 1 (Middle East Asia Minor 1, previously known as B biotype) and MED (Mediterranean, previously known as Q biotype) [6–8].

TYLCV has been detected not only in tomato but also in several other crops and ornamental plants including tobacco [9–11], common bean [12], corchorus [13], cowpea [14], eustoma [15], pepper [16–18], squash [19], and many weeds [20–29] in field surveys. These plants are suspected to be putative reservoirs for TYLCV survival and spread. However, only a few host plants have been tested and confirmed to be reservoirs for TYLCV by indoor transmission experiments [9, 16, 18, 25, 26, 29].

Transmission of geminiviruses by *B*. *tabaci* is the outcome of complex interactions between whiteflies, viruses, and host plants. The transmission efficiency varies depending on the combination of whitefly cryptic species, virus species (or isolate), and host plant species (or cultivar) [9, 16–18, 30]. Previous studies showed that TYLCV could be transmitted from virus-infected pepper (*Capsicum annuum* cv. Super Yellow GS) [16] and *C*. *annuum* cv. California Wonder [18] to healthy tomato and pepper plants, but it could not be transmitted from infected *C*. *annuum* cv. Cadia or cv. Dulce de España [17]. Therefore, different pepper cultivars may differ significantly in their role as virus reservoirs in the TYLCV epidemic.

As an economically important crop, hundreds of tobacco cultivars are cultivated in China. These cultivars are classified into several types, such as flue-cured tobacco, aromatic tobacco, and sun-cured tobacco, based on their differences in chemical composition, morphological and biological characters, and culturing or curing method. Previous studies showed that an aromatic tobacco cultivar (*Nicotiana tabacum* cv. Samsun) [9] and a sun-cured tobacco cultivar (*N*. *tabacum* cv. Havana 423) [11], which are mainly cultivated in Middle Asia and Cuba, respectively, were symptomless host plants of TYLCV. However, for the most important tobacco type, flue-cured tobacco, which accounts for the majority of tobacco production (~75% worldwide and over 90% in China, respectively) [31], little is known about the acquisition and transmission of TYLCV between this crop and tomato by whiteflies, and this knowledge is of vital importance to determine the role of flue-cured tobacco as a reservoir plant. In this study, we investigated the susceptibility of nine widely cultivated flue-cured tobacco cultivars by agrobacteria- (agro-) or whitefly-mediated inoculation. Subsequently, we studied the acquisition and transmission of TYLCV by *B*. *tabaci* MED by using infected tobacco as a virus source.

## Materials and methods

### Ethics statement

The samples we collected in field survey involved whitefly *Bemisia tabaci*, flue-cured tobacco and several other common plants. *B*. *tabaci* is a worldwide destructive agricultural pest insect, which is not included in the “List of Endangered and Protected Animals in China”. The tobacco tissues we collected were from a common flue-cured tobacco cultivar that is widely used for cigarette production. Therefore, field survey carried out during the years 2015–2017 did not involve any endangered or protected species, and no specific permits were required for this kind of regular pest survey. The owners of the tobacco and tomato field granted us permission to collect tobacco leaf samples, whiteflies, and other plants.

### Field survey

Tobacco leaf samples were collected from a tobacco field (34°51′56″N 115°35′41″E) approximately 500 m away from a tomato field (34°51′53″N 115°35′18″E) with a high incidence of TYLCV infection in Zhengzhou, Henan Province from 2015 to 2017(Table 1). Leaf samples of other plants were collected in or around the tomato field. Meanwhile, 20–50 adult whiteflies from each plant species were also collected and identified using the PCR-RFLP described by Qin et al. [32].

**Table 1:** Field survey of *Tomato yellow leaf curl virus* (TYLCV) in Zhengzhou, Henan province, China.

Family	Species	Infectivity (diagnosis by PCR)^a^	Symptom ^b^	Whitefly		Collection date
				Density ^c^	Cryptic species	
Apocynaceae	*Catharanthus roseus*	0% (0/5)	NS	+	MED	Aug/2017
Compositae	*EcIipta prostrata*	0% (0/5)	NS	++	MED	Aug/2016
	*Lactuca serriola*	0% (0/5)	NS	+	MED	Aug/2016
Euphorbiaceae	*Acalypha australis*	10% (1/10)	NS	+	MED	Aug/2015, Sept/2016
Fabaceae	*Purple Haricot*	0% (0/5)	NS	+	MED	Aug/2016
Labiatae	*Lagopsis supina*	0% (0/20)	NS	++	MED	Sept/2016
	*Perilla frutescens*	0% (0/5)	NS	+	MED	Aug/2017
Moraceae	*Broussonetia papyrifera*	0% (0/5)	NS	+	MED	Aug/2017
Solanaceae	*Nicotiana tabacum*	17% (5/30)	NS	+++	MED	Aug/2016, Aug/2017

^a^ Number of TYLCV infected plants/number of plants

^b^ NS: No symptoms

^c^ +, ++, +++ represent 1–5, 5–20, 21–50 whitefly adults in 10 cm^2^ leaf area, respectively

### Insects

Whiteflies in the field survey were identified as *B*. *tabaci* MED, a widespread *B*. *tabaci* cryptic species in China [33, 34]. *B*. *tabaci* MED adults collected from the tobacco field and confirmed to be virus-free by PCR were reared on cotton (*Gossypium hirsutum*. cv. Zhemian 1793), which is a non- host for TYLCV, in insect-proof cages for five generations. To obtain a large population of whiteflies, the whiteflies from cotton plants were then reared on healthy tobacco (*N*. *tabacum* cv. Cuibi 1) for two generations. Insect rearing was conducted in a separate room and PCR diagnosis of whiteflies and tobacco plants was performed every month to guarantee the virus-free status of those organisms. Environmental conditions for insect rearing and following experiments were 25±3°C, 50±10% humidity, and a12h L: 12h D photoperiod. One day to seven days old whitefly adults were used in this study.

### Transmission of TYLCV to tobacco plants by agro- or whitefly-mediated inoculation

Nine tobacco cultivars (4-5-true leaf stage) (Table 2) were injected with an infectious clone containing TYLCV genome (GenBank accession no. AM282874). For each cultivar, five plants (4–6 true leaf stage) were used. Meanwhile, five tobacco (*N*. *tabacum* cv. Cuibi 1) plants (4–6 true leaf stage) were inoculated at a density of 30 viruliferous whiteflies per plant by using clip-cages with an inoculation access period (IAP) of 48 h and then sprayed with spirotetramat (50 mg/L). The viruliferous whiteflies were obtained by releasing aviruliferous whiteflies onto a TYLCV-infected tomato (*Solanum lycopersicum* Mill. cv. Hezuo903) plant with an acquisition access period (AAP) of 48h [30]. Both whitefly and agro-inoculated tobacco plants were grown at 25±3°C, 50±10% humidity, and a12h L:12h D photoperiod in insect-proof cages. TYLCV infection of each plant was determined by symptom analysis and PCR 30 days after inoculation. TYLCV-infected tobacco (*N*. *tabacum* cv. Cuibi 1) obtained by agro-inoculation was used as a virus source in the experiments described below.

**Table 2:** Infection rate in tobacco plants after agro-inoculation.

Tobacco cultivar	Infectivity (diagnosis by PCR)^a^	Symptom^b^
Cuibi 1	100% (5/5)	NS
NC 89	100% (5/5)	NS
Qinyan 96	100% (5/5)	NS
Yunyan 99	100% (5/5)	NS
Yuyan 12	100% (5/5)	NS
Honghuadajinyuan	80% (4/5)	NS
K326	80% (4/5)	NS
Yunyan 87	60% (4/5)	NS
Zhongyan 100	40% (2/5)	NS

^a^ Number of TYLCV infected plants/number of plants inoculated

^b^ NS: no symptoms

### Acquisition of TYLCV DNA by whiteflies from infected tobacco plants

This experiment was done to determine the acquisition efficiency of TYLCV by MED whiteflies from infected tobacco. Approximately 300 whitefly adults were released onto two infected tobacco plants enclosed in an insect-proof cage. Thereafter, 10–15 adults were randomly collected at the designated AAP on the plants (Table 3). The collected insects were stored at -20°C. For each AAP, 10 insects were analyzed individually for TYLCV DNA acquisition by PCR.

**Table 3:** Efficiency of acquisition of TYLCV DNA from infected tobacco plants by *B*. *tabaci* MED.

Acquisition access period (h)^a^	% adults with TYLCV DNA
0.25	10
0.5	20
1	20
2	30
4	50
6	60
8	80
10	90
12	100
24	100
48	100

^a^ For each duration, 10 adults were analyzed

### Transmission of TYLCV from infected tobacco to tobacco and tomato plants by whiteflies

According to the result of acquisition experiment (Table 3), all whiteflies were found to have carried TYLCV 48 hours after they fed on TYLCV-infected plants. Therefore, approximately 600 whitefly adults were released onto two TYLCV- infected tobacco plants or two healthy tobacco plants for 48 h to obtain viruliferous or aviruliferous whiteflies, respectively. The tobacco *N*. *tabacum* cv. Cuibi 1 (4–6 true leaf stage) or the tomato *S*. *lycopersicum* Mill. cv. Hezuo903 (4–6 true leaf stage) plants were inoculated with TYLCV at densities of 1, 5, and 10 viruliferous individuals per plant by using clip-cages with an IAP of 48 h. The plants were sprayed with spirotetramat (50mg/L) to kill whitefly eggs or possible larvae and then grown at 25±3°C, 50±10% humidity, and a12h L:12h D photoperiod in insect-proof cages. Tobacco or tomato plants inoculated with aviruliferous whiteflies were used as negative control. For each inoculation, 20 plants of two hosts (ten plants for each) were used. Infection of plants by TYLCV was determined by both symptom analysis and PCR 30 days after inoculation. This experiment was repeated three times.

### Detection of TYLCV DNA by PCR

Total DNA from the leaves (second leaf from the top) from treated and control plants were extracted using method described by Xie *et al*. [35]. Total genomic DNA from a single whitefly was extracted using the method described by Qin *et al*. [32]. Primer pairs TYLCV-652 (5’-ATCGAAGCCCTGATATCCCCCGTGG-3’) and TYLCV-1340 (5’-CAGAGCAGTTGATCATG-3’) [36] were used to detect TYLCV DNA in plants and whiteflies. PCR reaction was carried out using the following conditions: preheating at 94°C for 3 min, then 30 cycles of 1 min at 94°C, 45 s at 52°C and 45 s at 72°C, followed by 72°C for 10 min.

## Results

### Field survey

TYLCV DNA was detected in tobacco and the weed *Acalypha australis* (Table 1). No visible symptom such as leaf curl or yellowing was observed on TYLCV positive tobacco or *A*. *australis* plants. Population densities of whiteflies on tobacco plants were significantly higher than those on other plant species (Table 1).

### Transmission of TYLCV to tobacco plants by agro- or whitefly-mediated inoculation

TYLCV was detected in both agro- and whitefly-mediated inoculated tobacco (*N*. *tabacum* cv. Cuibi 1) plants (S1 Fig). The infectivity varied from 40% to 100% among different tobacco cultivars (Table 2). No visible symptoms were developed on virus infected tobacco plants.

### Acquisition of TYLCV DNA by whiteflies from virus-infected tobacco plants

Percentage of whiteflies with TYLCV DNA increased with the increasing AAP to the infected tobacco plants (Table 3). The viral DNA was first detected in whiteflies with a 15 min AAP to infected plants, and reached 100% after a 12 h AAP to infected plants (Table 3).

### Transmission of TYLCV from virus infected tobacco to tobacco and tomato plants by whiteflies

TYLCV was successfully transmitted from infected tobacco plants to tobacco and tomato plants by whiteflies (Table 4). Typical symptoms of TYLCV infection were observed on virus infected tomato but not on tobacco plants. For both tobacco and tomato plants, the percentage of infected plants increased with increasing inoculation densities (Table 4). TYLCV was not detected in control plants inoculated with aviruliferous whiteflies.

**Table 4:** Transmission frequency of TYLCV by *B*. *tabaci* MED after an acquisition access period of 48 h on infected tobacco plants and an inoculation access period of 48 h on tobacco or tomato plants.

No. of whiteflies/plant	Replication	Infection of recipient plants with TYLCV following transmission ^a^	
		Tobacco	Tomato
1	1	0% (0/10)	10% (1/10)
	2	10% (1/10)	10% (1/10)
	3	10% (1/10)	20% (2/10)
5	1	30% (3/10)	30% (3/10)
	2	30% (3/10)	40% (4/10)
	3	40% (4/10)	30% (3/10)
10	1	80% (8/10)	80% (8/10)
	2	70% (7/10)	60% (6/10)
	3	70% (7/10)	80% (6/10)

^a^ Number of TYLCV infected plants/number of plants inoculated.

## Discussion

Previous studies have shown that TYLCV DNA can be detected in whiteflies with a minimum AAP of 5–30 min on infected tomato plants [30, 37–39]. Similarly, our data showed that TYLCV DNA was detected in whiteflies after a 15 min AAP on infected tobacco plants, suggesting TYLCV was capable of being readily acquired from infected tobacco plants by *B*. *tabaci* MED (15 min, 10% whitefly were found to be associated with TYLCV, Table 3).

**Fig 1:**
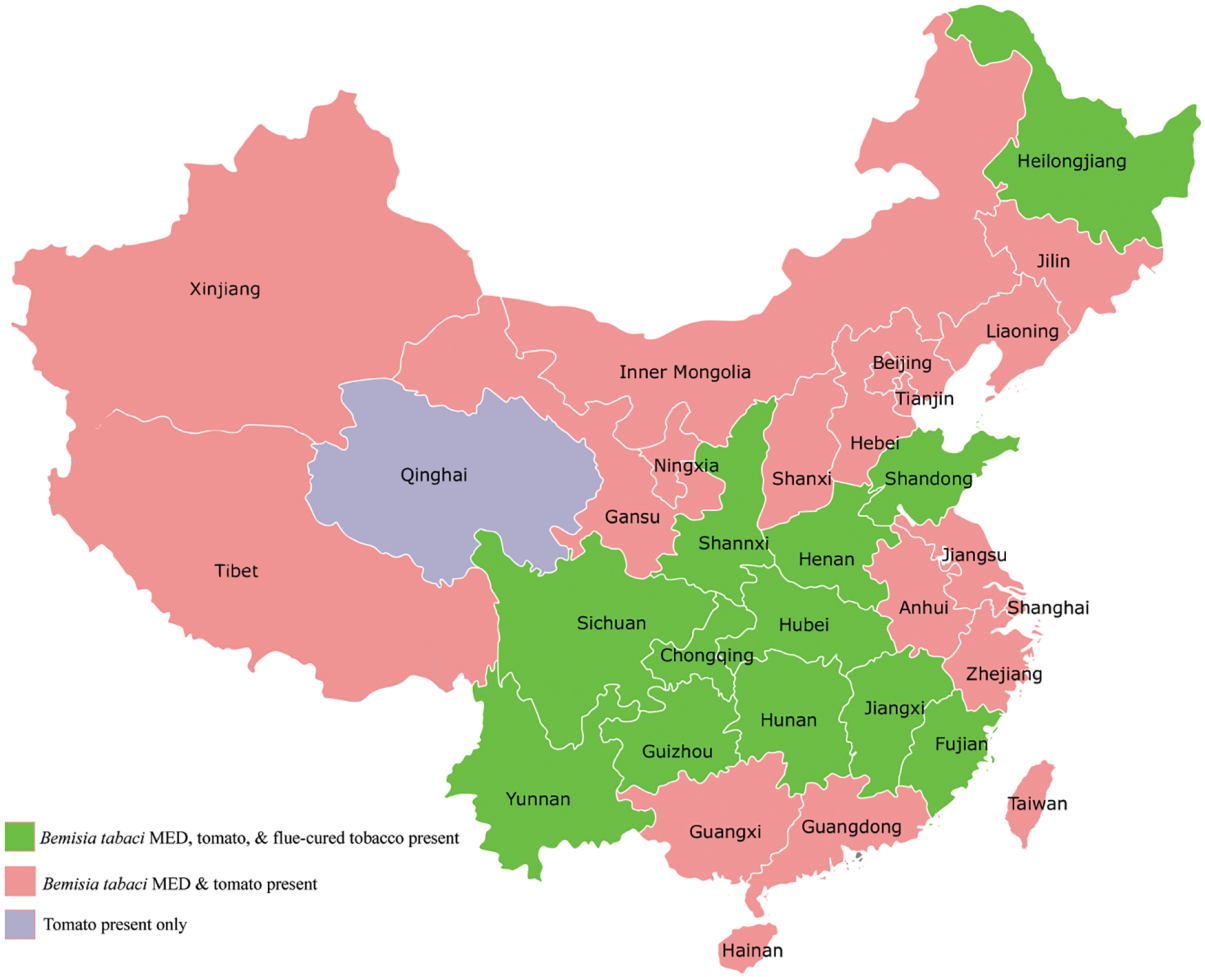
Growing areas of flue-cured tobacco and tomato, and the distribution of the invasive whitefly *Bemisia tabaci* MED in China. The map was generated based on the data from [33, 34, 41–45].

TYLCV was first detected in tomato plants in Shanghai, China in 2006 [40]. Since then, outbreaks of TYLCV epidemics have been reported in all the tomato production areas including eastern, central, and southwestern China [10, 30], which is largely overlapped the major flue-cured tobacco-growing areas in China (Fig 1). Since the cultivation of flue-cured tobacco and tomato in the same area is common in many tobacco-growing areas (especially in Henan, Shandong, and Hunan province) in China (Fig 1), tobacco may harbor both TYLCV and its vectors, whiteflies, after tomato harvest and may become the infection source for the next tomato-growing season. In this study, nine flue-cured tobacco cultivars that are widely cultivated in China were found to be infected by TYLCV through agro-inoculation. Transmission experiments demonstrated that TYLCV was effectively transmitted to healthy plants from infected tobacco plants by *B*. *tabaci* MED at inoculation densities of 1, 5, and 10 viruliferous whiteflies per plant with an IAP of 48 h. Therefore, we suggest separating the cultivation of tomato and tobacco to prevent continuous outbreaks of TYLCV epidemics in tomato fields, which usually cause a great loss of tomato production. If the cultivation of tomato and tobacco in the same area is inevitable, the cultivation system, isolation distance, and timing should be carefully considered to make effective management strategies for TYLCV control.

## Supporting information

S1 Fig Detection of TYLCV DNA in the tobacco (*N*. *tabacum* cv. Cuibi 1) plants after whitefly transmission. M. DNA marker; Lane 1, negative control; Lane 2, positive control; Lane 3–7, PCR products of 5 tobacco plants after virus inoculation by whiteflies.
(TIF)
