# Supplementary figures and images for: Flue-cured tobacco confirmed as a reservoir host plant for Tomato yellow leaf curl virus by agro-inoculation and Bemisia tabaci MED-mediated transmission
Source: PLoS One. 2017 Dec 22;12(12):e0190013. doi: 10.1371/journal.pone.0190013 (PMC5741240; doi:10.1371/journal.pone.0190013)

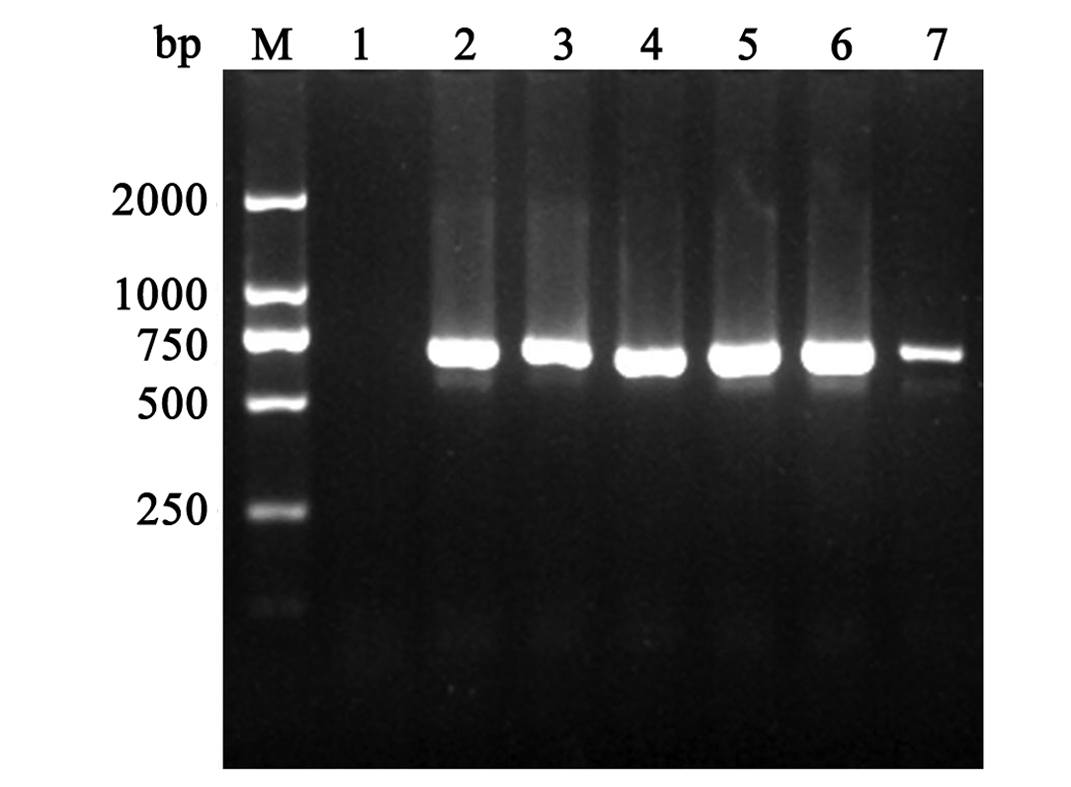

Supplement: S1 Fig — M. DNA marker; Lane 1, negative control; Lane 2, positive control; Lane 3–7, PCR products of 5 tobacco plants after virus inoculation by whiteflies. (TIF) [file pone.0190013.s001.tif]
